# Association between *TNFAIP3* nonsynonymous single-nucleotide polymorphism rs2230926 and chronic hepatitis B virus infection in a Chinese Han population

**DOI:** 10.1186/s12985-015-0268-6

**Published:** 2015-02-28

**Authors:** Pingping Zhang, Na Li, Qianqian Zhu, Fang Li, Cuiling Yang, Xiaoyan Zeng, Yi Lv, Zhihua Zhou, Qunying Han, Zhengwen Liu

**Affiliations:** Department of Infectious Diseases, First Affiliated Hospital, School of Medicine, Xi’an Jiaotong University, Xi’ an, 710061 Shaanxi Province China; Department of Laboratory Medicine, First Affiliated Hospital, School of Medicine, Xi’an Jiaotong University, Xi’ an, 710061 Shaanxi China; Department of Hepatobiliary Surgery, First Affiliated Hospital, School of Medicine, Xi’an Jiaotong University, No. 277 Yanta West Road, Xi’an, 710061 Shaanxi Province China; Institute of Advanced Surgical Technology and Engineering, Xi’an Jiaotong University, Xi’ an, 710061 Shaanxi China

**Keywords:** Hepatitis B virus, *TNFAIP3*, Polymorphism, Susceptibility, Clinical disease

## Abstract

**Background:**

Single-nucleotide polymorphisms (SNPs) in tumor necrosis factor alpha-inducible protein 3 (*TNFAIP3*) gene have been linked to inflammatory, immunological and malignant diseases. Hepatitis B virus (HBV) infection is characterized by immunopathogenesis. This study investigated the association of rs2230926, a nonsynonymous SNP in *TNFAIP3* gene, with chronic HBV infection.

**Methods:**

Four hundred and fifty-five patients with chronic HBV infection with clinical diseases of chronic hepatitis (n = 183), liver cirrhosis (n = 167) and hepatocellular carcinoma (n = 105), 92 HBV infection resolvers and 171 healthy controls were included. All subjects were of Chinese Han ethnicity. Genotyping of rs2230926 was carried out by polymerase chain reaction-restriction fragment length polymorphism method.

**Results:**

The gender and age between HBV patients, HBV infection resolvers and healthy controls had no statistical difference. The genotypes of rs2230926 in HBV patients, HBV infection resolvers and healthy controls are in Hardy-Weinberg equilibrium. The genotype and allele frequencies of *TNFAIP3* rs2230926 polymorphism between HBV patients, HBV infection resolvers and healthy controls had no significant difference. The genotype and allele frequencies of *TNFAIP3* rs2230926 polymorphism between HBV patients with chronic hepatitis, liver cirrhosis and hepatocellular carcinoma also showed no significant difference.

**Conclusions:**

The *TNFAIP3* rs2230926 polymorphism is not suggested to be associated with the susceptibility of chronic HBV infection or the progression of HBV-related diseases in this study. Replicative studies and studies in large control and HBV patient populations of different ethnicity by genotyping more polymorphisms in *TNFAIP3* gene are needed.

## Background

Hepatitis B virus (HBV) infection is a major cause of liver disease. Globally, two billion people have been infected with HBV, 360 million have chronic infection, and 600,000 die each year from HBV-related liver diseases including liver cirrhosis and hepatocellular carcinoma (HCC) [1]. The development and progress of HBV-associated liver disease is regarded as a result of the interplay between the virus and the host’s immune response [2,3]. The intensity of immuno-inflammatory responses of the hosts to various levels of the HBV viral replication characterizes the natural course of chronic HBV infection and has been extensively studied, showing that immunoinflammatory responses associated with various types of cells are involved in the course [4-9]. In particular, T cell exhaustion, which is characterized by a hierarchical and progressive loss of T-cell functions [10], is a significant feature of chronic HBV infection. Up-regulation of inhibitory molecules and down-regulation of effector function are characteristics of the exhaustion of HBV-specific T cells [11,12]. Because the excess of inhibitory molecules is among the critical factors driving T cell exhaustion in chronic HBV infection, blockade of these inhibitory molecules including programmed death-1 (PD-1), cytotoxic T-lymphocyte-associated antigen 4 (CTLA-4) and T-cell immunoglobulin domain and mucin domain-containing molecule-3 (Tim-3) has been demonstrated to reconstitute the potential of functional HBV-specific T cells [11,12]. Genetically, single-nucleotide polymorphisms (SNPs) in *PD1* [13], *CTLA4* [14] and *TIM3* [15] have also been demonstrated to be associated with the disease progression of chronic HBV infection. However, the role of these molecules in chronic HBV infection appeared to be non-redundant and the rescue of HBV T cell specificities by their blockade was incomplete and not all patients responded to these strategies, suggesting the possible role of other inhibitory molecules.

Tumor necrosis factor alpha-inducible protein 3 (TNFAIP3, also known as A20), a cytoplasmic zinc finger protein with ubiquitin-modifying activity, has been identified as a negatively regulating molecule that inhibits nuclear factor kappa-B (NF-κB) activity and tumor necrosis factor (TNF)-mediated programmed cell death [16-19]. Human genetic studies have linked polymorphisms in the *TNFAIP3* gene to inflammatory, autoimmune and malignant diseases [20]. Among the SNPs in *TNFAIP3* gene, rs2230926, a nonsynonymous common coding SNP in exon 3 of *TNFAIP3* which introduces the amino acid substitution of phenylalanine to cysteine at amino acid position 127 of the protein, has been functionally shown to possibly influence the mRNA expression of TNFAIP3 and the inhibitory activity of TNFAIP3 [21-23]. Furthermore, *TNFAIP3* rs2230926 polymorphism has been demonstrated to be significantly associated with systemic lupus erythematosus (SLE) among multiple ethnic populations [21,24-29] and other autoimmune diseases such as Sjögren’s syndrome, Crohn’s disease, psoriasis and rheumatoid arthritis [30,31].

The possible role of TNFAIP3 in chronic HBV infection remains largely unknown. Given the functional relevance, the common frequency and the strong association with immune-associated diseases of rs2230926 polymorphism in the *TNFAIP3* gene as well as the involvement of immunoregulatory and inflammatory responses in chronic HBV infection, the aim of this study was to examine the possible association between *TNFAIP3* rs2230926 polymorphism and chronic HBV infection in a Chinese Han population.

## Methods

### Patients and controls

Patients with chronic HBV infection were recruited from the First Affiliated Hospital, School of Medicine, Xi’an Jiaotong University, a tertiary hospital in the northwest China. All patients were tested to be negative for markers of hepatitis C virus (HCV) and human immunodeficiency virus (HIV). Coexistence of autoimmune, alcoholic or metabolic liver disease was also excluded. Patients who had never been treated with nucleos(t)ide analogues or interferon (IFN)-α were eligible for inclusion. Patients were diagnosed as a clinical disease of chronic hepatitis (CH), liver cirrhosis (LC) or HCC based on history of HBV infection, HBsAg/anti-HBs, HBeAg/anti-HBe and anti-HBc serostatus, HBV DNA level, biochemical liver function, α-fetoprotein (AFP) level, and ultrasonography and/or computerized tomography (CT)/ magnetic resonance imaging (MRI) as described previously [14]. Moreover, HBV infection resolvers and healthy individuals were recruited as controls. The resolution of HBV infection was based on normal liver biochemistries and seropositivity for anti-HBs and anti-HBc. Healthy controls were those who had normal liver biochemistries, no history of hepatitis B, and seropositivity for anti-HBs only or seronegativity for HBV markers. In total, 455 patients with chronic HBV infection [age, 41.8 ± 13.2 (18–77) years; male/female, 347/108] were enrolled. The clinical diagnoses of the patients were 183 chronic hepatitis, 167 liver cirrhosis and 105 HCC (Table 1). For comparison, 92 HBV infection resolvers [age, 44.3 ± 15.1 (18–78) years; male/female, 63/29] and 171 healthy controls [age, 42.7 ± 15.8 (19–76) years; male/female, 116/55] were also recruited (Table 1).

**Table 1:** **Demographics and Hardy-Weinberg equilibrium of the genotypes of rs2230926 G/T polymorphism in HBV patients, HBV infection resolvers and healthy controls and the clinical diagnosis of HBV patients**

	**Patients (** ***n*** **= 455)**	**Resolvers (** ***n*** **= 92)**	**Controls (** ***n*** **= 171)**	***P***
Sex (male/female)	347/108	63/29	116/55	0.06
Age [year, mean ± SD (range)]	41.8 ± 13.2 (18–77)	44.3 ± 15.1 (18–78)	42.7 ± 15.8 (19–76)	0.45
HWE (observed vs. expected, *P* value)	0.53	0.19	0.08	
Clinical diagnosis				
Chronic hepatitis	183			
Liver cirrhosis	167			
HCC	105			

HBV, hepatitis B virus; HWE, Hardy-Weinberg equilibrium; HCC, hepatocellular carcinoma.

### Determination of serum HBV markers and liver biochemistry

Serum HBV markers including HBsAg, anti-HBs, HBeAg, anti-HBe and anti-HBc were detected using ELISA from Beijing Wantai Biological Pharmacy (Beijing, China). Biochemical liver function including alanine aminotransferase (ALT) and aspartate aminotransferase (AST) levels (IU/L) was assayed on the Olympus AU5400 automatic biochemical analyzer (Olympus Corporation, Mishama, Japan). Serum HBV DNA levels (IU/ml) were quantitatively determined using hepatitis B virus fluorescence polymerase chain reaction diagnostic kit manufactured by Da An Gene Co., Ltd. of Sun Yat-Sen University (Guangzhou, China) according to the instruction. Serum AFP levels (ng/ml) were measured using automated Eleceyes (Roche Diagnostics, Mannheim, Germany).

### Genotyping of *TNFAIP3* rs2230926 polymorphism

Genomic DNA was extracted from EDTA-treated peripheral blood using TIANamp Genomic DNA Kit [Tiangen Biotech (Beijing) Co., Ltd., Beijing, China] according to manufacturer’s instruction. Genotyping of *TNFAIP3* rs2230926 G/T polymorphism was carried out by DNA amplifications with polymerase chain reaction (PCR) and specific primer sets, followed by the restriction fragment length polymorphism (RFLP) method. The primers used for amplification are: Forward: (12169–12188) 5′-CTCCTTTGCAGTTGGTGTCA-3′ and Reverse: (12698–12717) 5′-GCTTCGCTTAGCCAAATTCA-3′ [32], according to *TNFAIP3* sequence (NG_032761). The PCR was carried out in a volume of 25 μl reaction, containing 12.5 μl 2 × Taq plus PCR Mix (Xi’an Runde Biotechnology, Xi’an, China),1.0 μl (10 μM) forward primer, 1.0 μl (10 μM) reverse primer, 6 μl genomic DNA and 4.5 μl sterile double distilled water. The reaction mixture was pre-heated at 94°C for 5 min, then amplified 30 cycles using the following program: heated at 94°C for 30 s, annealing at 60°C for 30 s and extension at 72°C for 1 min, and lastly extended at 72°C for 10 min. The undigested PCR product has a length of 549 bp. The restriction fragments of the polymorphism were obtained using restriction endonuclease Fnu4HI [New England Biolabs (Beijing) Ltd., Beijing, China] according to the instruction of the manufacturer. The Fnu4HI has a restriction site on the PCR products with G allele (12486–12490, **T**CAGC > **G**CAGC). Digested fragments were analyzed by electrophoresis on 2% agarose gels stained with ethidium bromide. The genotypes of the polymorphism were determined according to the digestion patterns: genotype TT: a fragment of 549 bp, genotype GT: 3 fragments of 549 bp, 319 bp and 230 bp, respectively, and genotype GG: 2 fragments of 319 bp and 230 bp, respectively.

### Statistical analysis

Statistical analysis was performed by SPSS software version 16.0 (SPSS, Inc., Chicago, IL). The frequency of genotypes and alleles was determined by direct gene counting method. Hardy-Weinberg equilibrium of the polymorphism was tested with χ^2^ test. The association between polymorphism and disease status was tested using χ^2^ test for contingency tables or Fisher’s exact test where applicable [33]. Odds ratios (OR) and its 95% confidence interval (CI) were calculated to estimate the risk conferred by a particular genotype and allele. A *P* value < 0.05 was considered statistically significant.

### Ethical approval and consent

This study was approved by the Review Board of the First Affiliated Hospital, School of Medicine, Xi’an Jiaotong University and conducted in accordance with the Declaration of Helsinki. Written informed consent was obtained from all the participants for the publication of this report.

## Results

### Demographics and Hardy-Weinberg equilibrium of the genotypes of rs2230926 in the study subjects

The demographics of the study subjects and the clinical diagnosis of the HBV patients are presented in Table 1. All subjects were of Chinese Han ethnicity. The gender and age between HBV patients, HBV infection resolvers and healthy controls had no statistical difference (Table 1). The genotypes of rs2230926 in HBV patients, HBV infection resolvers and healthy controls are in Hardy-Weinberg equilibrium (*P* > 0. 05, Table 1).

### Genotype and allele frequencies of rs2230926 polymorphism in hepatitis B virus (HBV) patients, HBV infection resolvers and healthy controls

The genotype and allele frequencies of rs2230926 polymorphism in HBV patients, HBV infection resolvers and healthy controls are shown in Table 2. There were no significant differences in the genotype and allele frequencies of rs2230926 polymorphism between HBV patients, HBV infection resolvers and healthy controls (Table 2).

**Table 2:** **Genotype and allele frequencies of the rs2230926 G/T polymorphism in hepatitis B virus (HBV) patients, HBV infection resolvers and healthy controls**

	**Patients**	**Resolvers**	**Controls**	***P***	**Patients vs. resolvers**		**Patients vs. controls**		**Resolvers vs. controls**	
	**(** ***n*** **= 455)**	**(** ***n*** **= 92)**	**(** ***n*** **=171)**		***P***	**OR (95% CI)**	***P***	**OR (95% CI)**	***P***	**OR (95% CI)**
Genotype										
TT	429 (94.3)	84 (91.3)	164 (95.9)	0.31	0.28	0.64 (0.28-1.45)	0.42	1.42 (0.61-3.34)	0.13	2.23 (0.78-6.36)
GT	26 (5.7)	8 (8.7)	7 (4.1)							
Allele										
T	884 (97.1)	176 (95.7)	335 (98.0)	0.32	0.29	1.55 (0.69- 3.47)	0.43	0.71 (0.31-1.65)	0.13	0.46 (0.16-1.29)
G	26 (2.9)	8 (4.4)	7 (2.1)							

Data are presented as *n* (%).

### Genotype and allele frequencies of rs2230926 polymorphism in hepatitis B virus (HBV) patients with different clinical diagnosis

The genotype and allele frequencies of rs2230926 polymorphism in HBV patients with different clinical diagnosis are shown in Table 3. There were no significant differences in the genotype and allele frequencies of rs2230926 polymorphism between HBV patients with different clinical diagnosis (Table 3).

**Table 3:** **Genotype and allele frequencies of rs2230926 G/T polymorphism in hepatitis B virus (HBV) patients with different clinical diagnosis**

	**Chronic hepatitis**	**Cirrhosis**	**HCC**	***P***	**Chronic hepatitis vs. Cirrhosis**		**Chronic hepatitis vs. HCC**		**Cirrhosis vs. HCC**	
	**(** ***n*** **= 183)**	**(** ***n*** **= 167)**	**(** ***n*** **= 105)**		***P***	**OR (95% CI)**	***P***	**OR (95% CI)**	***P***	**OR (95% CI)**
Genotype										
TT	175 (95.6)	158 (94.6)	96 (91.4)	0.33	0.66	1.25 (0.47-3.31)	0.15	2.05 (0.77-5.49)	0.30	1.65 (0.63-4.29)
GT	8 (4.4)	9 (5.4)	9 (8.6)							
Allele										
T	358 (97. 8)	325 (97.3)	201 (95.7)	0.34	0.66	1.24 (0.47-3.25)	0.15	2.00 (0.76-5.28)	0.31	1.62 (0.63- 4.14)
G	8 (2. 2)	9 (2.7)	9 (4.3)							

Data are presented as *n* (%). HCC, hepatocellular carcinoma.

## Discussion

This study investigated the possible association of *TNFAIP3* nonsynonymous SNP rs2230926 with chronic HBV infection in Chinese Han population. The genotype and allele frequencies of rs2230926 polymorphism in the healthy control population of this study were: genotype TT, 95.91%; genotype GT, 4.09%; allele T, 97.95%; and allele G, 2.05%. Considering the minor allele, the allele G in this study was lower than other studies in Chinese Han populations (4.5%-5.9%) [27,34] and Korean population (5.3%) [35]. Although studies in Chinese Han population revealed very low frequency of genotype GG (0.091%-0.3%) [27,34], we did not detect genotype GG in both the control and patient populations and this is consistent with the findings in Korean population [35]. These results indicate that the rs2230926 polymorphism may vary greatly even in populations with genetic vicinity.

This study showed that the genotype and allele frequencies of *TNFAIP3* rs2230926 polymorphism between HBV patients, HBV infection resolvers and healthy controls and among HBV patients with different clinical diagnosis had no significant differences, suggesting that this polymorphism might not contribute to the susceptibility of chronic HBV infection or the progression of HBV-related diseases. Previous studies showed that this polymorphism is associated with other diseases especially autoimmune disorders [21,24-31]. It is therefore suggested that the significance of this genetic polymorphism might be restricted to specific diseases.

It is noteworthy that recent studies proposed that a pair of tandem polymorphic dinucleotides (rs148314165, rs200820567, referred to as TT > A) located in the genomic DNA 30 kb telomeric of *TNFAIP3* may be the most likely candidate variants responsible for association with SLE and the TT > A risk alleles are carried on a risk haplotype that is associated with hypomorphic expression of TNFAIP3 transcripts and protein and the TT > A risk variants attenuate TNFAIP3 expression through inefficient delivery of NF-kB to the TNFAIP3 promoter [36,37], evidencing the critical functional role of TT > A in the genetic predisposition to autoimmune diseases. Therefore, considering the relevance of TNFAIP3 molecule to the regulation of NF-κB activity and TNF-mediated programmed cell death [16-19] and the involvement of NF-κB activity and programmed cell death in HBV infection [38-40], the possible predisposition effect of this TT > A polymorphism in chronic HBV infection deserves investigation in future studies.

It should be noted that this study has limitations. The findings were preliminary results from a limited number of HBV patients and controls of Chinese Han population. The study only analyzed the association of one polymorphism with cross-sectional diseases in chronic HBV infection. The lack of replicative patient and control populations also compromised the probative value of the findings. Therefore, further studies in large patient and control populations of different ethnicity with longitudinal follow-up of the patients by genotyping more polymorphisms in *TNFAIP3* gene are needed to clarify the possible effect of *TNFAIP3* polymorphisms on chronic HBV infection and HBV-related diseases.
